# A Machine Learning Approach for PLGA Nanoparticles in Antiviral Drug Delivery

**DOI:** 10.3390/pharmaceutics15020495

**Published:** 2023-02-02

**Authors:** Labiba Noorain, Vu Nguyen, Hae-Won Kim, Linh T. B. Nguyen

**Affiliations:** 1 Department of Pharmaceutics, School of Pharmacy, University College London, London WC1N 1AX, UK; 2 Machine Learning Research Group, Eagle House, University of Oxford, Oxford OX2 6ED, UK; 3 UCL Eastman-Korea Dental Medicine Innovation Centre, Dankook University, Cheonan 31116, Republic of Korea; 4 Institute of Tissue Regeneration Engineering, Dankook University, Cheonan 31116, Republic of Korea; 5 BK21 NBM Global Research Centre for Regenerative Medicine, Dankook University, Cheonan 31116, Republic of Korea; 6 Eastman Dental Institute, University College London, Royal Free Hospital, Rowland Hill Street, London NW3 2PF, UK

**Keywords:** nanoparticles, machine learning, antiviral, PLGA

## Abstract

In recent years, nanoparticles have been highly investigated in the laboratory. However, only a few laboratory discoveries have been translated into clinical practice. These findings in the laboratory are limited by trial-and-error methods to determine the optimum formulation for successful drug delivery. A new paradigm is required to ease the translation of lab discoveries to clinical practice. Due to their previous success in antiviral activity, it is vital to accelerate the discovery of novel drugs to treat and manage viruses. Machine learning is a subfield of artificial intelligence and consists of computer algorithms which are improved through experience. It can generate predictions from data inputs via an algorithm which includes a method built from inputs and outputs. Combining nanotherapeutics and well-established machine-learning algorithms can simplify antiviral-drug development systems by automating the analysis. Other relationships in bio-pharmaceutical networks would eventually aid in reaching a complex goal very easily. From previous laboratory experiments, data can be extracted and input into machine learning algorithms to generate predictions. In this study, poly (lactic-co-glycolic acid) (PLGA) nanoparticles were investigated in antiviral drug delivery. Data was extracted from research articles on nanoparticle size, polydispersity index, drug loading capacity and encapsulation efficiency. The Gaussian Process, a form of machine learning algorithm, could be applied to this data to generate graphs with predictions of the datasets. The Gaussian Process is a probabilistic machine learning model which defines a prior over function. The mean and variance of the data can be calculated via matrix multiplications, leading to the formation of prediction graphs—the graphs generated in this study which could be used for the discovery of novel antiviral drugs. The drug load and encapsulation efficiency of a nanoparticle with a specific size can be predicted using these graphs. This could eliminate the trial-and-error discovery method and save laboratory time and ease efficiency.

## 1. Introduction

Traditional pharmaceutical drug development processes depend on trial-and-error methods, which are time-consuming and costly, and depend on finding the optimum formulation in the laboratory, making them challenging. In addition, they are limited by experimental conditions such as high equipment supplies, controlled experimental environments and practical experience [1,2,3]. Hence, there is a crucial need to design a new paradigm for time and performance efficiency for nanomaterials science.

A large amount of experimental data is currently available and can be used in machine learning algorithms to generate predictions. Machine learning can become a promising path to accelerating nanomaterials design and applications using predictions for future antiviral drug design [4,5]. In this present study, we employed data pertaining to poly (lactic-co-glycolic acid) (PLGA) nanoparticles to predict the drug loading capacity and encapsulation efficiency of antiviral agents. The findings of these predictions could aid the design of novel antiviral drugs and allow discoveries to be made more efficiently. The first-generation paradigm requires the featuring of raw data in descriptors and building models from the descriptors. The second generation paradigm is unique, as it eliminates human feature engineering as the models can be made from automated feature engineering [6]. Machine learning can combine experimental and theoretical methods for future perspectives. The integration of this machine learning method into drug development pipelines would decrease the time and cost of production. To date, the study of machine learning approaches for predicting the properties of antiviral agents has not yet been conducted, making this study a novel area of research. Machine learning approaches have excelled in other fields, and more research needs to be done on machine learning for drug discovery and design due to the potential success it can offer.

This study’s predictions will focus on nanoparticle size, polydispersity index (PDI), drug loading capacity and encapsulation efficiency. Particle size is a vital factor in the design of nanomaterials in antiviral activity. Nanomaterials ranging in size from 100–300 nm have shown to be successful in avoiding the liver and spleen, which metabolises nanoparticles and reduces circulation time. Particle geometry and surface characteristics also play a crucial role, as they allow specific cellular targeting [7]. Nanoparticle size can be changed via alternating the solution conditions, polymer concentration, manufacturing, drug loading, and the release of drugs [8]. The manipulation of nanoparticle size has been investigated to enhance bioavailability, increase cellular uptake, and improve drug delivery efficiency. By controlling the size of the nanoparticles, targeted drug delivery can be achieved [9,10]. PDI is the measurement of a sample’s heterogeneity based on nanoparticle size. Nanoparticles can have a large PDI due to a large size distribution or agglomeration of the sample [11]. A low PDI (close to 0) means that the sample is narrowly dispersed, which is the goal, as a high polydispersity (up to 1) can result in an assortment of nanoparticles with fluctuating loading capacities, lower physical stability, and different release profiles [12]. The drug load is the ratio of drugs to the nanoparticle. The encapsulation efficiency is the percentage of medicine effectively captured into the nanoparticle. 

The aims of these studies are to generate prediction graphs of PLGA for future antiviral drug design and discovery. The first objective is to gather data on polymer and metal nanoparticles in antiviral drug delivery by thoroughly searching the literature and data mining. The second objective that brings novelty to this study is to use data from PLGA nanoparticles and analyse the data to predict drug-loading capacity and the entrapment efficiency of antiviral agents, information not reported previously. The project uses the Gaussian Process, a machine learning algorithm, to provide new sets of drug loading and encapsulation efficiency predictions according to nanoparticle size and polydispersity index data [13,14]. This could eliminate the trial-and-error discovery method, save laboratory time, and ease efficiency.

## 2. Materials and Methods

**Figure 1:**
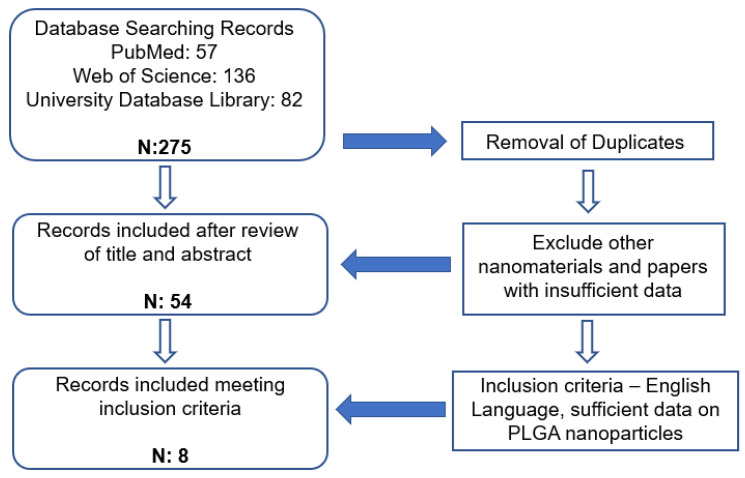
PLGA Nanoparticles Data Extraction Algorithm.

A search of the national and international publications was undertaken using PubMed, Web of Science and the UCL Database Library with the search term ‘nanoparticles in antiviral activity’. The present study involved an extensive examination of a variety of nanomaterials, with a particular focus on PLGA. Subsequently, an investigation of PLGA nanoparticles with varying ratios was carried out. For PLGA nanoparticles, the following terms were explored: ‘PLGA nanoparticles in antiviral activity’ and ‘PLGA nanomaterials against viruses’. These searches from three different databases produced 275 publications. These were put together and examined to remove duplicates. This resulted in 54 papers which were reviewed based on the title and abstract to discover only literature about PLGA nanoparticles, and these review papers were removed. Finally, the literature papers were further studied to find sufficient data on PLGA nanoparticles, as indicated in Figure 1. Data was found on nanoparticle size, polydispersity index (PDI), drug load and encapsulation efficiency from eight research articles. The equation for drug load and encapsulation efficiency calculation is displayed in Equations (1) and (2).

(1) $$\mathrm{Drug}\;\mathrm{Loading}\;(\%)=\frac{Weight\;of\;drug\;in\;nanoparticles}{Weight\;of\;total\;nanoparticle}\;\times \;100$$

(2) $$\mathrm{Encapsulation}\;\mathrm{Efficiency}\;(\%)=\frac{Weight\;of\;drug\;in\;nanoparticle}{Initial\;weight\;of\;blank\;drug}\;\times \;100$$

Review papers were removed from the results; the literature consisted of papers in English and research papers only. The search took place in July 2020 and includes papers from 2010 onward. Before the machine learning analysis, the gathered data was prepared. The dataset was checked for missing data and then organised in numerical order according to particle size.

The data analysis took place via the Gaussian Process [13,14]. The Gaussian Process (GP) is a probabilistic machine learning model which defines a prior over function. After observing some function values, it can be converted into posterior over functions given the data. In this context, the inference of continuous function values is widely known as GP regression. In the setting of this study, the data input includes a two-dimensional vector. To perform prediction at all possible input values, the Gaussian posterior prediction distribution was estimated. Particularly for such Gaussian distributions, there is a need to estimate both the mean and the variance. The computation for the mean and variance involves matrix multiplications and inversions, as described in [13,14]. Using GP modelling, the desirable property that similar input tends to have similar output was taken advantage of. Thus, it establishes a statistical correlation between the output and the input across two dimensions. Although the study has considered two-dimensional input, it is noted that the Gaussian process can be generalised to handle more than two input dimensions, as shown in recent applications with GP [14,15].

## 3. Results

### 3.1. PLGA 50:50 Nanoparticles

The search of the database resulted in the finding of eight papers with data on PLGA nanoparticles.

**Figure 2:**
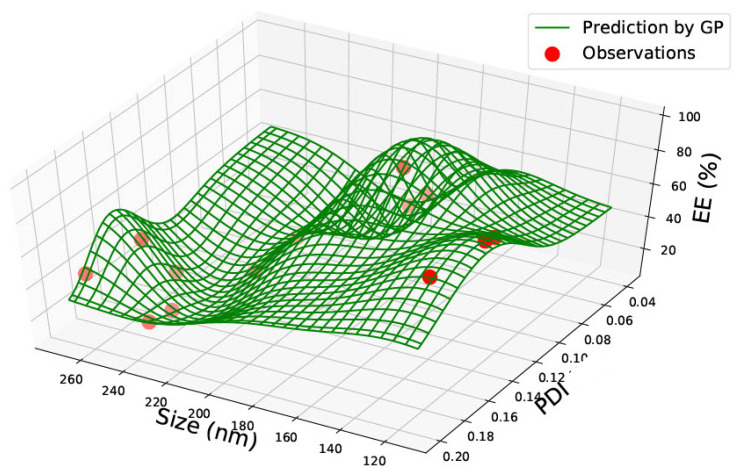
PLGA 50:50—graph showing size (nm), Polydispersity Index (PDI) and Encapsulation Efficiency (EE%).

**Figure 3:**
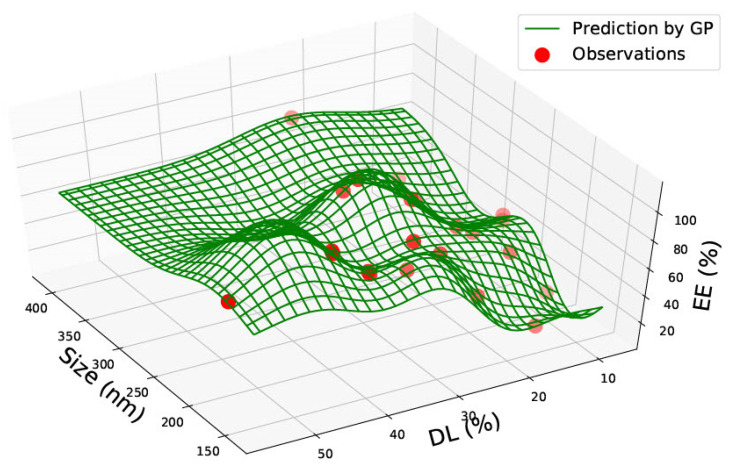
PLGA 50:50—graph showing size (nm), % Drug Load (DL) and Encapsulation Efficiency (EE%).

Table 1 represents the accumulated sixty-two data points gathered from the literature on PLGA 50:50 nanoparticles. Data was found on size, polydispersity index, drug load, and encapsulation efficiency. The data found was not consistent, and very rarely was data found on all four categories. However, the combination of the data allowed two prediction graphs to be generated, as shown in Figure 2 and Figure 3.

Figure 2 represents a graph with size, PDI and encapsulation efficiency for PLGA 50:50 nanoparticles. The red circles represent the data found in the literature, and the green lines represent the predictions made using the Gaussian Process. Areas where the green lines are high on the axis indicate that the prediction probability is higher, creating a wave-like structure. The graph represents a non-linear relationship between the three components, and no correlation can be found within the data. The encapsulation efficiency fluctuates with various nanoparticle sizes. At a particle size of approximately 200 nm, the highest encapsulation efficiency of approximately 60% with a low PDI between 0.02–0.04 can be observed. The encapsulation efficiency also varies and reaches a little over 50% at approximately 260 nm, with a higher PDI of 0.18. Hence, this proves that the encapsulation efficiency fluctuates and a direct correlation between size and encapsulation efficiency cannot be assumed. Therefore, the graph would be most beneficial when predicting future antiviral drug designs where the encapsulation efficiency can be estimated based on the size and PDI of the desired nanomaterial. For example, in Figure 2, at a size of 240 nm and PDI of 0.18, the encapsulation efficiency was very low (between 0–20%). A researcher could use this information and create a nanoparticle size of 200 nm instead, where encapsulation efficiency is predicted to be higher.

In Figure 3, the graph represents the size, drug load and encapsulation efficiency for PLGA 50:50 nanoparticles. No linear relationship can be seen between the three factors. The drug load is seen to be low in the graph, except between 300–350 nm, where it is seen to be the highest at approximately 40%, with an encapsulation efficiency of roughly 40%. Similar to Figure 2, high entrapment efficiency is seen between 200–300 nm. The two graphs in Figure 2 and Figure 3 could be used in combination to predict drug load and encapsulation efficiency for PLGA 50:50 nanoparticles. For example, Figure 2 predicted that entrapment efficiency is higher for a 200 nm particle size. However, Figure 3 predicts that the drug load would be low (between 10–20%) at 200 nm. Therefore, a different particle size could be chosen based on the study’s requirements and the nanoparticle’s function.

### 3.2. PLGA 65:15 Nanoparticles

**Figure 4:**
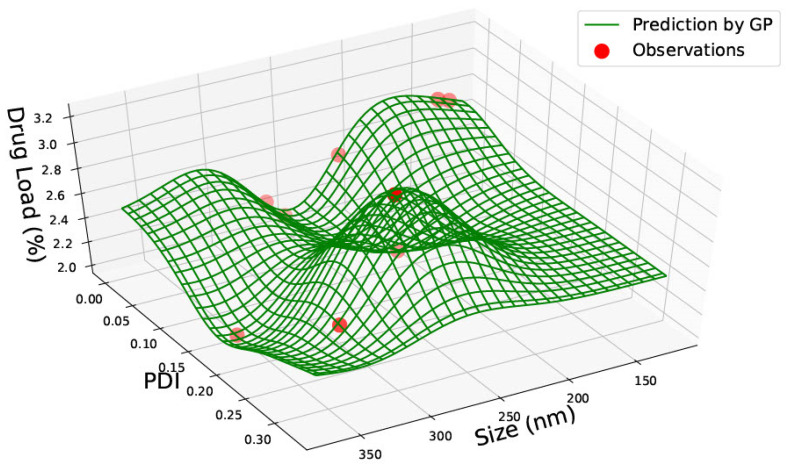
PLGA 65:35—graph with size (nm), Polydispersity Index (PDI) and Drug Load (%DL).

**Figure 5:**
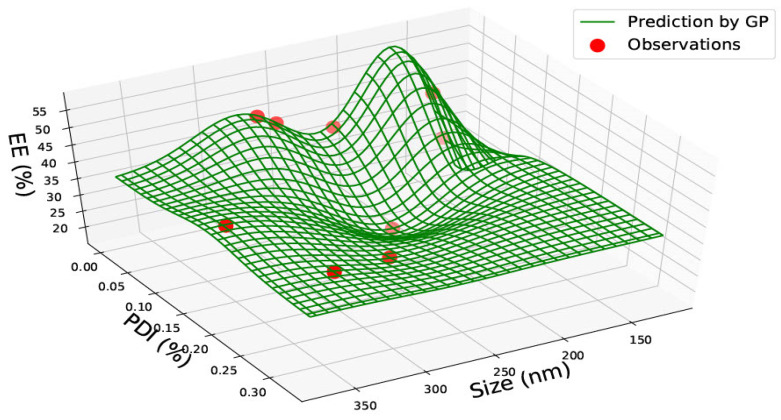
PLGA 65:35—graph with size (nm), Polydispersity Index (PDI) and Encapsulation Efficiency (EE%).

**Figure 6:**
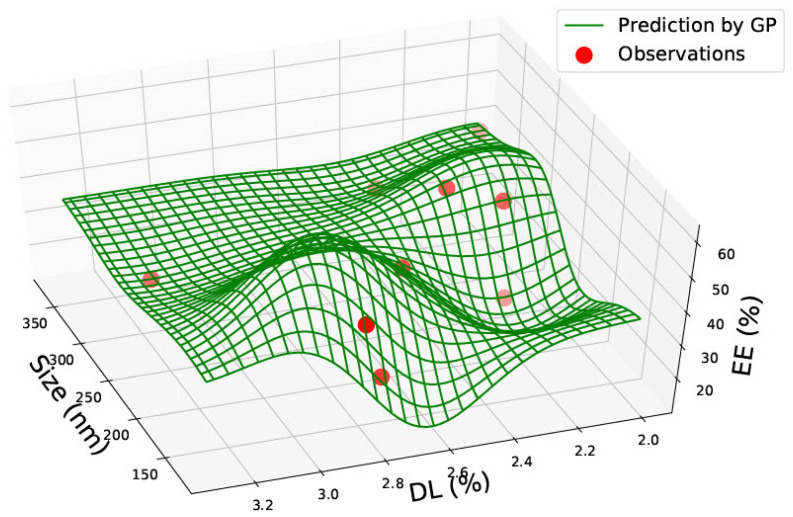
PLGA 65:35—graph with size (nm), Drug Load (DL%) and Encapsulation Efficiency (EE%).

Data was gathered on PLGA 65:15, 75:25 and 85:15 nanoparticles from one single paper [23] with nine data points. The data for PLGA 65:15 is represented in Table 2. Figure 4, Figure 5 and Figure 6 illustrate the three predicted graphs from the data gathered from Table 2, representing the size, polydispersity index, drug load and encapsulation efficiency of PLGA 65:15 nanoparticles. There is a smaller amount of data available for this ratio than the PLGA 50:50 ratio. However, this data is consistent, where all four information factors are available for each data point.

Figure 4 represents graphs with size, PDI and drug load. Similar to PLGA 50:50, no correlation between the three categories can be seen. The data is scarcely distributed along the plot; however, the predictions exhibit that the highest drug load of 2.8% can be achieved with smaller nanoparticles (approximately 150 nm) and low PDI (between 0–0.05). The drug load is slightly high (2.2%) for a nanomaterial size of approximately 200 nm and a PDI of 0.15. However, the drug load is seen to be lowest (below 2%) at a nanoparticle size of about 350 nm and PDI of roughly 0.15, suggesting that drug load is not dependent on nanomaterial size or PDI.

Figure 5 represents the predicted graph of PLGA 65:35 nanomaterial data with size, PDI, and encapsulation efficiency. The predictions exhibit the highest encapsulation efficiency of approximately 55% with lower particle-sized nanomaterials of 150–200 nm and low PDI between 0–0.05. The encapsulation efficiency is also slightly high at 45%, with a nanomaterial size between 250–300 nm and a low PDI. In contrast to this, the encapsulation efficiency is predicted to be at its lowest of between 20–25% with nanomaterial sizes of 200–250 nm and a slightly higher PDI of 0.1. This suggests that there is no relation between size and encapsulation efficiency.

Figure 6 shows a graph with size, drug load and encapsulation efficiency for PLGA 65:15 nanoparticles. The predictions show that the highest encapsulation efficiency of approximately 50% exists for a nanoparticle size of 150–250 nm, where the drug load is also highest at 2.8%. Encapsulation efficiency is also high for nanoparticle sizes of 300–350 nm. However, the drug load is low here at less than 2%. The predictions can combine the three Figure 4, Figure 5 and Figure 6 and be altered based on the needs of the experiment in question. Each graph could also be used to cross-reference the other, as they contain at least two common denominators.

### 3.3. PLGA 75:25 Nanoparticles

**Figure 7:**
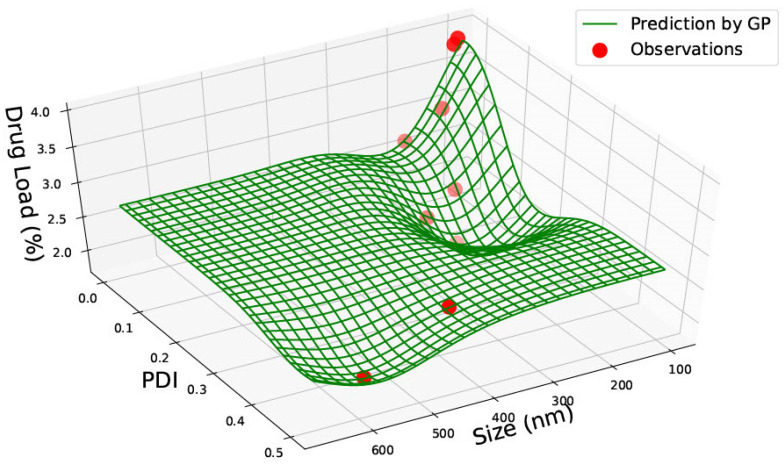
PLGA 75:25—Size (nm), Polydispersity Index (PDI) and Drug Load (DL%).

**Figure 8:**
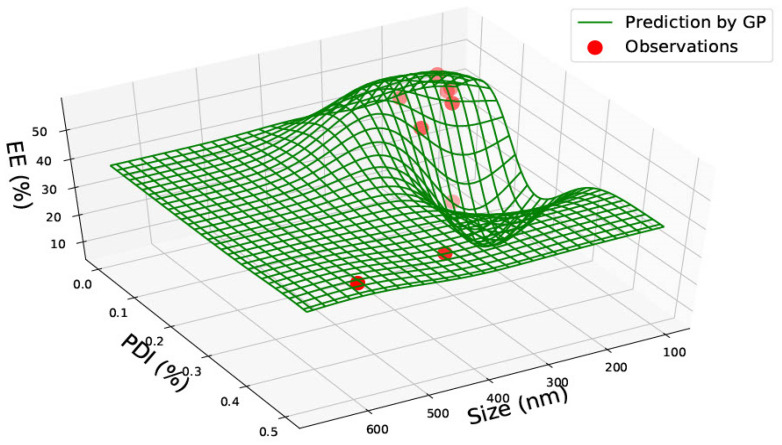
PLGA 75:25—size (nm), Polydispersity Index (PDI) and Encapsulation Efficiency (EE%).

**Figure 9:**
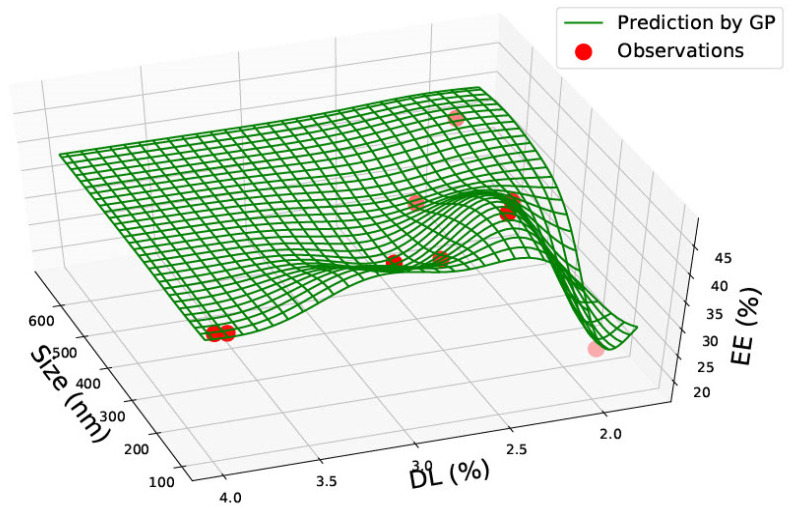
PLGA 75:25—graph representing size (nm), Drug Load (DL%) and Encapsulation Efficiency (EE%).

The data gathered from article [23] on PLGA 75:25 nanoparticles are represented in Table 3. Figure 7, Figure 8 and Figure 9 are prediction graphs generated from Table 3, which demonstrates data gathered on PLGA 75:25 nanoparticles with size, PDI, Drug load and encapsulation efficiency.

Figure 7 represents PLGA 75:25 nanoparticle data with size, PDI and drug load. It was seen that the highest drug load of approximately 4% was for the smaller-sized nanoparticles of 100 nm and PDI between 0–0.1. The lowest drug load of less than 2% was seen in two different nanomaterial sizes: one at approximately 200 nm with a PDI of 0.2 and the other between 500–600 nm with a higher PDI of 0.4. This does not represent any connection between size and drug load. However, the data heavily lies between 100–200 nm, and the limited data between 300–600 nm nanomaterial sizes are insufficient for prediction via GP. Therefore, predictions may be inaccurate in the 300–600 nm range.

In Figure 8, the graph represents PLGA 75:25 nanoparticles with size, PDI and encapsulation efficiency. The graph predicts that a smaller nanoparticle size between 100–200 nm has a lower PDI (between 0–0.1) and a high encapsulation efficiency of approximately 40%. The lowest encapsulation efficiency of less than 10% is also predicted for 100–200 nm, with a higher PDI of 0.2. This suggests that there may be a correlation between encapsulation efficiency and PDI; however, this cannot be confirmed due to insufficient data between 300–600 nm.

In Figure 9, the graph shows predictions for PLGA 75:25 nanoparticles with size, drug load and encapsulation efficiency. Similar to Figure 7 and Figure 8, drug load and encapsulation efficiency are predicted to be higher for smaller nanoparticles. Here, an outlier lies at the largest nanoparticle size (600 nm), where the predictions are not clear due to the scarcity of data. Instead of combining the graphs, the predictions would be more precise with regard to drug load and entrapment efficiency on separate graphs where PDI data is also available. Figure 9 can be used for confirmation purposes.

### 3.4. PLGA 85:15 Nanoparticles

**Figure 10:**
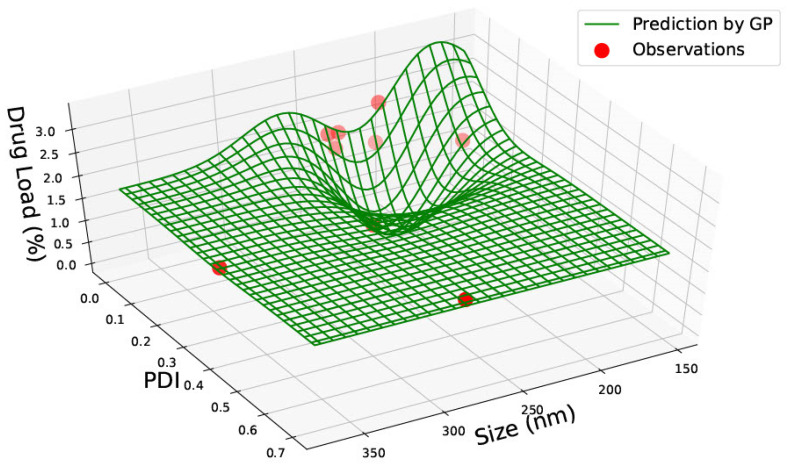
PLGA 85:15—graph representing size (nm), Polydispersity Index (PDI) and Drug Load (DL%).

**Figure 11:**
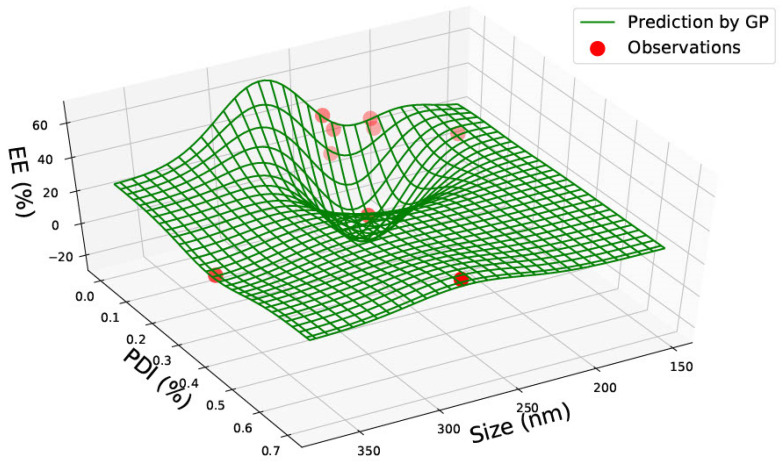
PLGA 85:15—graph representing size (nm), Polydispersity Index (PDI) and Encapsulation Efficiency (EE%).

**Figure 12:**
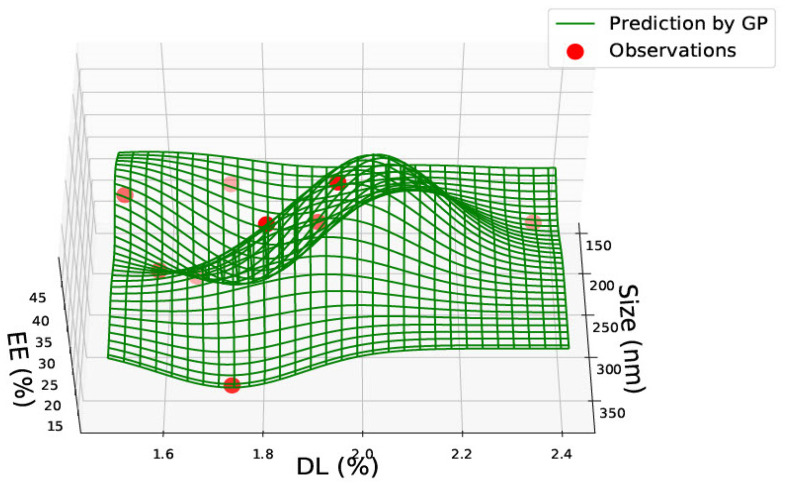
PLGA 85:15—graph representing size (nm), Drug Load (DL%) and Encapsulation Efficiency (EE%).

The data gathered from article [23], on PLGA 85:15 nanoparticles, are represented in Table 4. Figure 10, Figure 11 and Figure 12 are prediction graphs generated from Table 4, representing PLGA 85:15 nanoparticles with data on size, PDI, drug load and encapsulation efficiency. Three sets of graphs were generated from this data.

In Figure 10, the graph represents data on size, PDI and drug load. The graph estimates that the drug load is highest with the smallest sized nanoparticle of 150 nm and a low PDI of 0–0.1. The drug load is also lowest between 200–250 nm with a PDI of 0.1–0.2. The graph has two outliers, one between 200–250 nm and one between 300–350 nm. Similar to Figure 7 and Figure 8, there is insufficient data in these ranges for GP predictions.

Figure 11 demonstrates a graph of PLGA 85:15 nanoparticles with size, PDI and encapsulation efficiency. The highest encapsulation efficiency of approximately 60% is predicted for a nanoparticle size between 250–300 nm with low PDI between 0–0.1. The lowest encapsulation efficiency is predicted between nanoparticle sizes of 200–250 nm, with a slightly higher PDI value of 0.1–0.2. The encapsulation efficiency axis is seen to be below 0 in Figure 11; however, Table 4 does not exhibit any data points below 0. Therefore, GP has predicted the encapsulation efficiency to be close to 0 for nanomaterials within the 200–250 nm size range.

In Figure 12, the graph predicts size, drug load and encapsulation efficiency data. The encapsulation efficiency is highest at approximately 200 nm, and the drug load is highest at 150 nm. The drug load is lowest at approximately 200 nm. The predictions in Figure 10, Figure 11 and Figure 12 have given similar results in each graph, suggesting the accuracy of the predictions based on the combination of the data set. These three figures could be beneficial for predictions. For example, if a high entrapment efficiency is required for a specific experiment, Figure 12 can be used, which would show that the nanoparticle size should be between 150–200 nm. Confirming this in Figure 11 would give the same size prediction and provide PDI information. In this case, a low PDI is needed, which would suggest that the researcher control experimental conditions to aim for a low PDI.

## 4. Discussion

### 4.1. PLGA Nanoparticles

For PLGA 50:50 nanoparticles, data were extracted from seven papers as follows: the first paper [16] was an investigation of nelfinavir mesylate (NFV), an antiviral drug for the treatment of Acquired Immunodeficiency Syndrome (AIDS). NFV is known to have poor bioavailability and a short half-life, leading to clinical limitations. The study aimed to produce NFV-loaded PLGA nanoparticles to increase solubility and bioavailability and allow sustained release. The NPs were assessed according to particle size, zeta potential, morphology, drug content, encapsulation efficiency and dissolution studies. In vivo studies in rabbits demonstrated that bioavailability was enhanced 4.94-fold, drug release was sustained for 24 h, and half-life was increased compared to NFV suspension. Five batches of nanoparticles were prepared with varied PLGA concentrations, but there were constant drug concentrations where the rise in polymer concentration led to an upsurge in size. The batch with the highest drug loading and entrapment efficiency was chosen for further study. However, the data exhibited by the other nanoparticles were beneficial in this study.

The second paper chosen for PLGA 50:50 nanoparticles [17] investigated griffithsin (GRFT) for the treatment of human immunodeficiency virus (HIV-1). PH-responsive fibres comprised of PLGA or methoxypolyethylene glycol-b-PLGA (mPEG-PLGA) were produced with GRFT loaded. It is designed to release the drug under certain pH conditions investigated by various ratios of PLGA to the drug. mPEG-PLGA illustrated high GRFT loading and successful pH-dependent release against HIV-1. The fibres indicate a pH-dependent release for at least 72 h. The data on size in this study is the diameter of the fibres.

The next paper [18] chosen was based on lamivudine (LMV), an antiretroviral drug encapsulated into PLGA nanoparticles and conjugated with Lactosaminated-Human Serum Albumin (L-HSA) peptide. The conjugation resulted in a 2.17-fold rise in cellular uptake and 3.84 times extended retention. This can make the conjugated nanoparticles a promising target for the liver. Twenty various formulations were investigated with different polymer concentrations, and the optimum formulation was chosen for further study.

The fourth article, with data on PLGA 50:50 nanoparticles [19], investigated acyclovir-loaded mucoadhesive PLGA nanoparticles for the treatment of herpes. Using polymer-loaded nanoparticles enables the drug to offer sustained release over a prolonged period. Drug to polymer ratio and surfactant concentration was varied, altering particle size and % drug release. In vivo studies demonstrated that 57.71–78.31% of the drug was released in 32 h, showing sustained release.

The next chosen article, [20], combined two antiretrovirals, GRFT and dapivirine (DPV), to prevent HIV. This was possible due to the drugs having separate physicochemical properties and being able to target the fusion and reverse transcription of HIV replication specifically. Several batches of nanoparticles were manufactured and assessed for particle size, drug release, cytotoxicity, cellular uptake and in vitro bioactivity. PLGA NPs were approximately 180–200 nm and were effectively encapsulated with GRFT (45%) and DPV (70%). Nanoparticles showed no signs of toxicity and sustained bioactivity in a cell-based assay. Four different nanoparticles were prepared: a placebo, two nanoparticles with a drug encapsulated in each, and the fourth nanoparticle with both drugs encapsulated. Data were selected for the latter three nanoparticles of this study.

In the sixth paper selected [21], LMV-loaded PLGA nanoparticles were formulated. Similar properties as the above studies were investigated to discover the optimum formulation. In vitro studies of LMV-loaded NPs illustrated a prolonged release of approximately 144 h. The optimum formulation was found to be stable in the gastrointestinal tract for up to 24 h. Studies also demonstrated the upgraded bioavailability of LMV. Five different formulations of varying particle sizes were collected and investigated for this study.

The last paper was chosen for PLGA 50:50 nanoparticles [22], which investigated PLGA nanoparticles with NFV and the histone deacetylase inhibitor suberoylanilide hydroxamic acid (SAHA). The loaded nanoparticles were shown to target infected CD4^+^ T-cells and obstruct the HIV viral spread. Three different nanoparticle sizes were investigated for the presence and absence of SAHA, which was included in this study. The gathered data from seven articles led to 62 data points represented in Table 1. Figure 7 and Figure 8 are the two graphs generated from this data on size, PDI, drug load and encapsulation efficiency.

For PLGA 65:15, 75:25 and 85:15 nanoparticles, data were taken for one paper [23]. This article was a study of three different prodrugs of ganciclovir (GCV) distributed in a PLGA-PEG-PLGA polymer gel for the treatment of herpes simplex virus type 1 (HSV-1) induced viral corneal keratitis. No toxicity was observed in in vitro studies. The research showed that prodrugs loaded with PLGA nanoparticles spread in thermosensitive gels and can be a promising sustained-release drug delivery system. Various ratios of polymers and prodrugs were tested to produce the nine different nanoparticles.

All graphs generated have red circles as observations and green lines representing the GP predictions. Areas of high green waves represent areas of prediction probability depending on the position of the green lines.

According to Equations (1) and (2), the drug load is the percentage of drug in the nanoparticle, and encapsulation efficiency is the amount of drug present in the nanoparticle compared to the initial amount of drug taken. Therefore, based on theory, it cannot be assumed that drug load and encapsulation efficiency should be correlated. In PLGA 65:35 nanoparticles, drug load and encapsulation efficiency are seen to be high between 150–200 nm, suggesting that there could be a correlation for this ratio. However, it must be considered that the data here is taken from one single paper with the same drug. Therefore, the correlation seen may be due to the prodrug of GCV and may be unique to the properties of this drug. No correlation with size was seen.

In PLGA 75:25, nanoparticles correlation between encapsulation efficiency and PDI can be seen where lower PDI predicts higher encapsulation efficiency. However, due to the lack of data on particle sizes of 300–600 nm, this is hard to conclude from this graph.

In PLGA 85:15 and 50:50 nanoparticles, no correlations were observed. PLGA 50:50 nanoparticles graphs (Figure 2 and Figure 3) consist of the most data, sixty-two data points gathered from seven research articles. All graphs in this study could be highly valuable for a novel design, as the prediction could save valuable time and give an idea of which nanoparticle size should be chosen based on what drug load and encapsulation are predicted via the GP method. The predictions can be made use of depending on the requirements of the research. For example, based on the requirement of drug load and encapsulation efficiency, a suitable nanomaterial size can be found from the graph manufactured.

It is significant to note that, although predictions can be made based on the graphs and data available, experimental conditions may deviate from the result. The addition of different drugs may increase or decrease the loading capacity and encapsulation efficiency. Nine different antiviral drugs were investigated in the gathered PLGA nanoparticle data. Different drugs can alter nanoparticle size; however, these graphs can indicate the successful loading capacities and entrapment efficiencies in previous studies and aid in the first step of the research and skip the trial-and-error steps required.

### 4.2. Current Research in this Field

The use of machine learning in material sciences has been applied to other fields, as well as nanomedicine. The algorithmic learning of models can vastly speed up the system’s state space. The characterisation and design of nanoparticles can have advantages compared with machine learning methods. However, combinations of machine learning with experimental design can also offer additional benefits. Active machine learning uses techniques such as reinforcement learning to select only the most successful candidates for testing. This approach reduces the time taken for trial-and-error methods and has already gained success in clinical trial investigations for cancer treatment [24]. Other research has taken place with the combination of machine learning and nanomedicine, as outlined below.

Machine learning methods may also offer predictive analysis of protein surroundings with the knowledge of protein biophysiochemical characteristics, nanomaterial size, surface charge and solution ionic strength [25].

The size of the nanoparticles is determined at the early stages of drug design. It influences stability, surface area, in vivo behaviour and cellular uptake. Within the 1–100 nm size range, the nanoparticles dispersion in solution is governed by the surface charge attraction and repulsion between particles and steric effects [26]. A study on tumour targeting based on the enhanced permeability and retention effect showed better results with a particle size of 100–150 nm. The smaller particle size of 30 nm nanoparticles was quickly washed out from the body, and larger particle sizes of approximately 300 nm were gathered in the spleen and liver [27].

Another study investigated the features needed for polymer nanoparticle prediction using an Artificial Neural Network (ANN) algorithm. Fifty-one samples were used with four data inputs: the quantity of drug, polymer concentration, solvent ratio and mixing rate. This ANN algorithm was used to generate a method to predict the size of the polymer-based nanoparticles. This study found that the polymer concentration was one of the most vital features for determining the particle size [28]. A separate study predicted the drug load with the machine learning algorithm. The variables included were molecular weight, the ratio of polymer to drug and the number of blocks per polymer. The ratio of the polymer was critical for estimations to be generated [29].

The studies above researched the components separately, where the best parts were discovered via machine learning. In this research, in contrast, size, PDI, drug load and entrapment efficiencies were found in combination, which has not yet been completed.

Spherical nucleic acids (SNAs) were investigated in a separate study as cancer vaccine candidates. The study aimed to describe a methodology for discovering structure-activity relationships and design guidelines for SNAs. Several steps were taken to reach this goal. The point of interest was the use of machine learning for quantitively modelling the SNA immune activation and determining the lowest number of SNAs required for an optimum structure-activity relationship [30,31]. The study demonstrates machine learning prospects via data modelling to predict the activity of SNA. Supervised machine learning models were applied to automatically predict the immune activity of data generated on a selection of antigens and their positions. This paper demonstrates that machine learning can be used successfully to generate predictions of nanomaterial activity. The novelty of this research limits further studies to verify predicted data. However, similar to the research of this project, the predictions from this research can be used successfully to ease novel drug design and discovery.

## 5. Conclusions

This study focused on the use of data from existing literature to make predictions with the help of machine learning. Machine learning is a subfield of artificial intelligence that can generate predictions based on data sets. It uses algorithms and develops methods to provide an output. Nanomaterials in antiviral activity were the focus of this study based on the current need for antiviral medicine for novel viruses. Although many drug candidates are available for various viruses, they are limited, and many manage the symptoms of the virus rather than the virus itself. Nanomaterials can be a promising lead for the delivery of antiviral drugs due their benefits such as large surface area and nano size. More specifically, the study concentrated on PLGA, a biocompatible polymer nanoparticle. PLGA was chosen based on its biodegradability, acceptance by the FDA and high loading capacities. PLGA nanoparticles also demonstrate antiviral activity via different mechanisms in various viruses.

The data from previous experiments can create predictions via machine learning. Machine learning allows predictions to be generated via models and functions. The graphs generated in this study were predicted via the Gaussian Process, a non-parametric approach. The data of PLGA were extracted from the literature. Libraries such as Web of Science and PubMed were searched, and eight articles for PLGA were narrowed down with inclusion and exclusion criteria. Twelve sets of graphs were generated with the use of the Gaussian Process by estimating the mean and variance of the data. A statistical correlation was established between the data inputs and outputs to generate predictions.

Two-dimensional graphs were created for PLGA 50:50, 65:15, 75:25 and 85:15 nanoparticles with data inputs of particle size (size range from 110 nm to 1630 nm), PDI (range from 0.005 to 0.679), drug load (range from 1.52% to 94.10%) and entrapment efficiency (range from 16.6% to 98.7%). In general, it was observed that smaller size has smaller PDI with varying drug load entrapment efficiency. A non-linear relationship was found between the drug load and entrapment efficiency. The graphs generated can be used in future antiviral drug discovery to predict aspects such as drug loading capacity and encapsulation efficiencies of the nanomaterial. One of the biggest limitations of this study was the availability of existing data to carry out predictions. The graphs are better represented with well-curated datasets; however, the limited data available in the graphs generated also have limitations. The results in this manuscript could provide ideas to predict the choice of drugs and, thus, this could save valuable time and avoid the use of trial-and-error to generate a successful nanoparticle. Therefore, machine learning could revolutionise novel drugs and allow them to be used clinically.

## Figures and Tables

**Table 1:** PLGA 50:50 nanoparticle data extracted from the literature.

PLGA 50:50				
Size (nm)	PDI	%DL	EE%	Reference
118	0.14		84	[16,17,18,19,20,21,22]
119	0.13		80	
125	0.17		78	
140.3		17.50	27.50	
167		23	46	
171.8			30.72	
174.2		55.84	84.30	
180.3		36.75	77.50	
182.7			34.49	
184.3	0.06		45.90	
185.3		36	72	
186.6	0.08		70.10	
188.8	0.07		40.70	
188.9		40.61	91.24	
192.5		29.43	83.41	
192.8		10	20	
195.9		25.30	67.75	
198.1		39.81	88.19	
203.2		29	58	
204		14.50	51.30	
220.6			33.25	
221	0.10		30.28	
223.4	0.13		25.37	
230.7			42.18	
232.7			40.19	
234.2	0.20		28.25	
236		18.70	62.30	
236.7	0.18		21.88	
239		16	53.40	
244.3			46.64	
248.5			40.18	
250.1	0.15		25.71	
251.2		23.55	81.95	
256.3			39.27	
256.9			51.64	
258	0.16	9.62	49.90	
258.6			41.57	
259.4			37.76	
259.4			41.85	
260.7			49.46	
263		29.60	98.70	
268.7	0.19	8.34	40.27	
272.4		30.64	88.34	
274.3			47.25	
275.6		20.31	67.95	
276.9			46.81	
281.4			53.44	
284.2			51.64	
292.4			58.54	
299.8			60.91	
311.1			57.57	
336		16.20	54	
407		24	80	
740		80.16		
810		80.09		
870		84.12		
914		86.26		
1107		80.59		
1210		89.90		
1420		88.12		
1580		93.70		
1630		94.10		

**Table 2:** PLGA 65:35.

PLGA 65:35				
Size (nm)	PDI	%DL	EE%	Reference
134.7	0.005	2.76	27.6	[23]
143	0.005	2.79	41.8	
215.7	0.005	2.54	38.1	
223.6	0.123	2.2	22	
256.4	0.005	2.15	42.9	
269.5	0.005	2.3	46	
273.5	0.233	3.2	32	
323.8	0.260	2.41	36.1	
356.3	0.16	2.02	40.4	

**Table 3:** Data Extracted PLGA 75:25.

PLGA 75:25				
Size (nm)	PDI	%DL	EE%	Reference
110.6	0.005	3.94	39.4	[23]
116.6	0.005	3.87	38.7	
134.1	0.005	3.02	45.3	
181.8	0.112	2.39	47.7	
198	0.007	2.69	40.4	
205.6	0.155	1.82	18.2	
251.4	0.14	2.26	45.2	
401	0.422	2.52	37.8	
569.4	0.467	2.07	41.3	

**Table 4:** Data of PLGA 85:15.

PLGA 85:15				
Size (nm)	PDI	%DL	EE%	Reference
173.6	0.115	1.73	25.9	[23]
207.8	0.005	2.35	23.5	
210.6	0.009	1.52	30.4	
233.5	0.005	1.91	28.6	
237.8	0.018	1.66	16.6	
240.8	0.009	1.95	38.9	
266.6	0.315	1.59	23.8	
275.5	0.679	1.81	36.2	
367.5	0.353	1.74	17.4	

## Data Availability

Not applicable.
